# Instructional Design for Accelerated Macrocognitive Expertise in the Baseball Workplace

**DOI:** 10.3389/fpsyg.2016.00292

**Published:** 2016-03-02

**Authors:** Peter J. Fadde

**Affiliations:** Department of Curriculum and Instruction, Southern Illinois UniversityCarbondale, IL, USA

**Keywords:** perceptual-cognitive, pitch recognition, baseball, macrocognition, expertise

## Abstract

The goal of accelerating expertise can leave researchers and trainers in human factors, naturalistic decision making, sport science, and expertise studies concerned about seemingly insufficient application of expert performance theories, findings and methods for training macrocognitive aspects of human performance. Video-occlusion methods perfected by sports expertise researchers have great instructional utility, in some cases offering an effective and inexpensive alternative to high-fidelity simulation. A key problem for instructional designers seems to be that expertise research done in laboratory and field settings doesn't get adequately translated into workplace training. Therefore, this article presents a framework for better linkage of expertise research/training across laboratory, field, and workplace settings. It also uses a case study to trace the development and implementation of a macrocognitive training program in the very challenging workplace of the baseball batters' box. This training, which was embedded for a full season in a college baseball team, targeted the perceptual-cognitive skill of pitch recognition that allows expert batters to circumvent limitations of human reaction time in order to hit a 90 mile-per-hour slider. While baseball batting has few analogous skills outside of sports, the instructional design principles of the training program developed to improve batting have wider applicability and implications. Its core operational principle, supported by information processing models but challenged by ecological models, decouples the perception-action link for targeted part-task training of the perception component, in much the same way that motor components routinely are isolated to leverage instructional efficiencies. After targeted perceptual training, perception and action were recoupled via transfer-appropriate tasks inspired by *in situ* research tasks. Using NCAA published statistics as performance measures, the cooperating team improved from middling performance to first in their conference in Runs Scored and team Batting Average. This case suggests that, beyond the usual considerations of effectiveness and efficiency, there are four challenges to embedded training in the workplace setting —namely: duration, curriculum, limited resources, and buy in. In the case reported here, and potentially in many domains beyond sports, part-task perceptual-cognitive training can improve targeted macrocognitive skills and thereby improve full-skill performance.

## Introduction

Sport has long been considered a productive test bed for research on expert performance and training that can potentially accelerate the expertise of performers in military domains (Ward et al., 2008), and other contexts that require macrocognition (defined as cognitive adjustments to performance complexity, *cf*. Klein, 2010). Macrocognitive skills such as anticipation and rapid decision making (Eccles et al., 2008) can potentially be accelerated using *expertise-based training* (XBT) that draws upon the theories, findings, and methods of expertise research in order to design training programs that can efficiently and effectively train expertise in workplace settings (Fadde, 2009a, 2013). XBT focuses on part-task training of cognitive subskills, such as the recognition component of Klein's (1998) model of recognition-primed decision making (Fadde, 2009b). XBT was largely developed in the realm of high-performance sports but also has been applied to accelerating expertise in domains as disparate as classroom teaching (Fadde and Sullivan, 2013), online masters' programs (Tokmak et al., 2013), nursing education (Razer et al., 2015), and peer academic advising (Blair, 2015).

For readers who understand and appreciate expert performance in baseball (or perhaps cricket), this case study provides a deep dive into the pitcher-batter matchup that is at the heart of the sport. For others, the primary points of interest relate to designing training programs that not only apply expert performance research to the task of accelerating expertise but also present research opportunities. Importantly, research design takes a distinctly secondary role to workplace constraints in training-based research. What training-based research projects can offer to the expertise research community are, first, satisfaction with successful implementation of research and, second, insights from fit-in-field modifications that can suggest new basic research questions.

**Figure 1:**
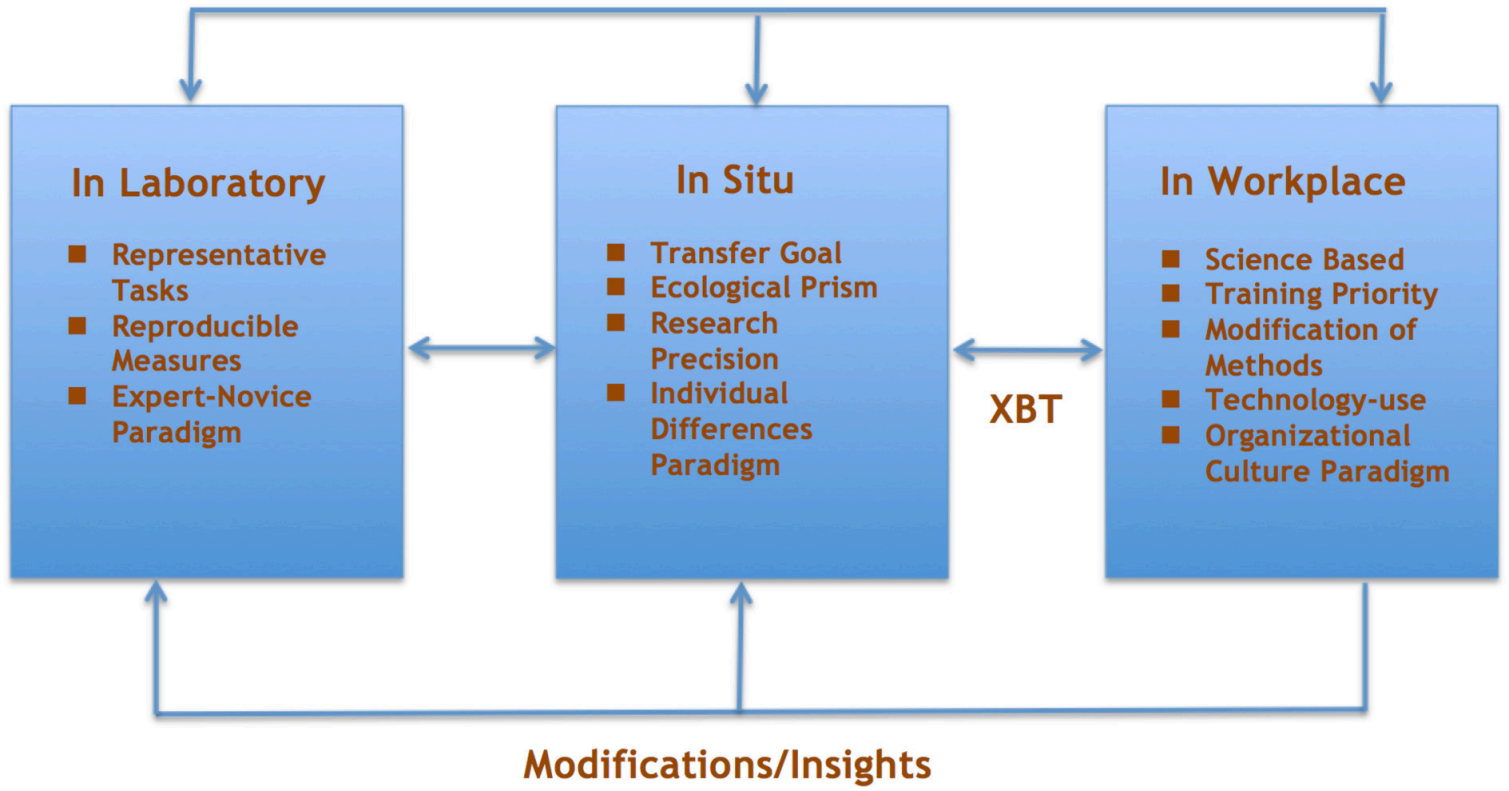
**Settings for expert performance research**.

The three settings for expertise research and training shown in Figure 1 are adapted from a three-stage expert performance model proposed by Williams and Ericsson (2005). Replacing *stages* with *settings* in the model emphasizes continuing and iterative processes rather than linear relationships.

“Ultimately, if the expert performance approach has validity, it should be demonstrable through the development of skill-sensitive training…to high levels of performance more quickly” (Charness and Tuffiash, 2008, p. 427). This article argues for and demonstrates an approach that, in the hands of professional instructional designers, military trainers, corporate designers-by-assignment, or human factors engineers, makes connections between expert performance research and expertise-based training. The case study then demonstrates adapting expert performance models and methods to training the perceptual-cognitive skill of *pitch recognition* that underlies one of the most extreme of human performances, hitting a pitched baseball traveling at speeds over 90 miles-per-hour and moving in unexpected directions.

### Transitioning expertise research to expertise training

Chief among the expertise research methods that have been successfully repurposed for expertise-based training is *temporal occlusion* in which subjects are shown film or video clips depicting a participant's view of an opponent, such as a baseball pitcher, cricket bowler, or tennis server. The film or video image is edited to black (occluded) at various points in the opponent's motion or ensuing ball flight. The representative task given to subjects or trainees is to identify the type of pitch or serve and sometimes predict where the ball will end up in the striking zone of a receiving player. The subject or trainee may respond verbally, by ticking an answer sheet, or even by making a realistic motion such as stepping to her backhand or forehand side to indicate serve location (Williams and Grant, 1999). Though occlusion points may vary across studies and sports, researchers, and trainers in this area agree that athletes can train their perceptual abilities by subtracting visual information during training. Most use video as the medium they rely on for training perceptual skills.

Not every researcher agrees that performance can be decoupled into smaller, more trainable cognitive units. Advocates of ecological dynamics (Davids et al., 2013) and direct perception (Bootsma and Harvey, 1997) argue strongly that decoupling the perception-action link in ballistic striking skills changes the behavior so that it can't be considered a truly representative task. Indeed, an entirely different visual response system, the dorsal stream, seems to be involved when a perception is intertwined with action rather than when perception is separated from action and therefore engages the ventral stream (Farrow and Abernethy, 2003). Distinct camps represent *predictive control* that holds a cognitive-information processing view supporting a pre-action perception stage, and a *prospective control* view based on Gibson's ecological approach to perception that loathes taking perception out of the context of actor and environment (Gray, 2009).

An information processing-based model has more utility from an instructional design perspective because it supports decoupling of the perception-action link for isolated and efficient training. Part-task training is generally more efficient than whole-task training strategies (for example, immersive simulations) that are supported by ecological views. While part-task training makes sense to baseball coaches who have long trained the mechanical components of batting in part-task ways, development of perceptual-cognitive or macrocognitive skills often is assumed to come only with substantial and varied authentic or simulated experience.

Temporal occlusion as a part-task perceptual training method in sports dates to Haskins' (1965) study that trained intermediate tennis players to recognize opponents' ground strokes. Although it predates articulation of the expert performance approach, Haskins' project shows how long the bones of occlusion training have been in place. As an *in situ* pre-test she filmed subjects returning groundstrokes from an opponent and counted frames of film between the opponent contacting the ball and the subject contacting the ball as a measure of response time. After multiple film-occlusion training sessions, subjects (college students) returned to the court and demonstrated a statistically significant improvement in response times. Haskins had not only created an occlusion-based training task but also devised an *in situ* pre/post-test that was ecologically valid for testing transfer of training gains to performance.

In the sport of baseball, Burroughs (1984) used video-occlusion to train pitch recognition as the perceptual-cognitive component of batting. Burroughs also devised an *in situ* occlusion device to test transfer of laboratory-based learning, which will be discussed further in the baseball training case study. *In situ* tasks not only are used to validate video-occlusion methods but also are used to study the relationship of perception and coordinated motor actions (e.g., Abernethy, 1984; Müller and Abernethy, 2006, 2012, 2014). For training purposes, adding *in situ* tasks may make up for the lack of ecological validity in typical video-occlusion laboratory tasks while also leveraging the precision and efficiency of tasks designed to reveal and measure the perceptual skills that underlie the extraordinarily rapid decision making of skilled athletes in many fast-action sports (Williams and Ward, 2003). Expert-novice studies typically do four things to reveal sources of expert advantage:

1. Identify critical perceptual-cognitive subskills of performance.
2. Devise representative tasks that target identified subskills and that are repeatable and readily measurable.
3. Test and compare highly skilled and less-skilled performers (expert-novice paradigm) to verify that performance on the task differentiates skill groups.
4. Iteratively add and subtract perceptual information or processing time to locate the boundaries of expert advantage.

Expert-novice studies reveal perceptual-cognitive skills that are critical to expert performance, and they also calibrate the representative tasks and methods used. These precisely defined testing tasks can become extremely efficient and effective training tasks, especially when presented in drill-and-practice format with immediate feedback and progressive difficulty (Alessi and Trollip, 2000). The progression from testing expert advantage to training expert advantage can be viewed in the context of baseball, particularly in the performance skill of batting and its perceptual-cognitive subskill of pitch recognition. In a model expert-novice study, Paull and Glencross (1997) compared the performance of more-skilled and less-skilled Australian professional baseball players on a video-occlusion task that involved identifying the type of pitch (fastball or curveball) being thrown by video pitchers. Pitches were occluded at a variety of points before, at, and after the moment-of-release of the pitch. Paull and Glencross identified which occlusion conditions were most predictive of expert-novice differences and Fadde (2006) used these occlusion points in generating video-occlusion items for a pitch recognition training project. Fadde also added instructional design value by creating Pitch Type, Pitch Location (Known Type), Pitch Location (Unknown Type), and Zone Hitting drills. Drills were edited onto separate videotapes, which were segmented by pitcher and occlusion condition.

**Figure 2:**
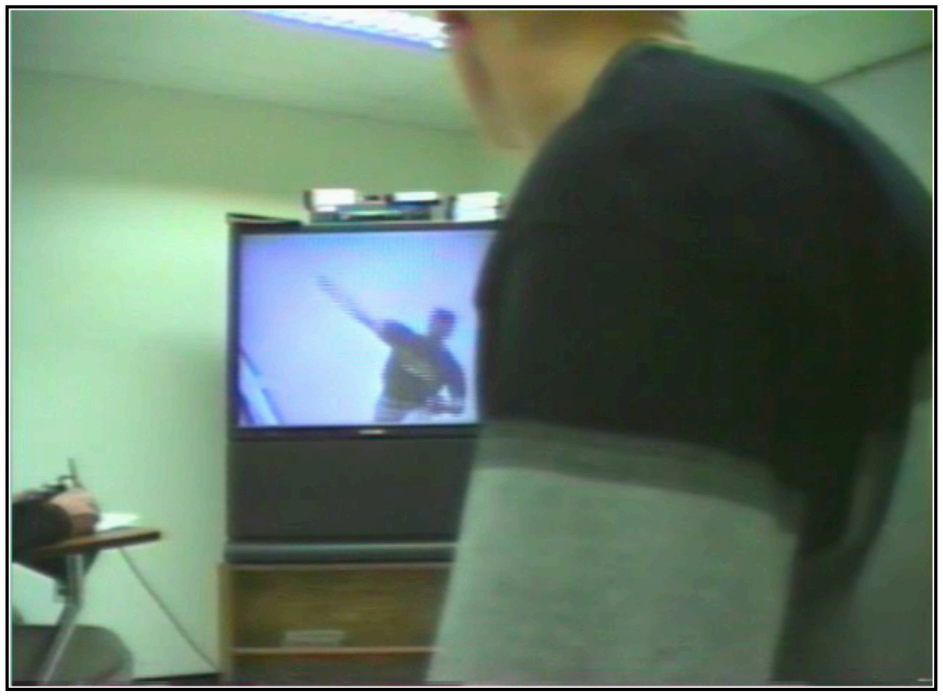
**Video-Occlusion in lab (1999)**.

As shown in Figure 2, a researcher/trainer conducting video-occlusion training would select a drill video, play a video pitch, record the player/trainee's verbal input (e.g., “Fastball” or “Strike”), provide immediate and corrective verbal feedback, and play the next video pitch. After completing all of the pitches in a drill, the researcher/trainer told the player his score on the drill. The player could choose to continue with the same drill video, viewing a different pitcher. The player could also view the same pitcher but at a more difficult occlusion point or choose a different video drill.

### Research-based training of pitch recognition

Video-occlusion tasks presented in a drill-and-practice instructional format have also been programmed into a sophisticated computer-based pitch recognition training application (Axon Sports, 2015). The *Axon Sports* computer program increases the fidelity level of video-occlusion training by using a 65-inch touch-screen video monitor for display. However, the *Axon Sports* program maintains the part-task recognition-only training approach rather than opting to simulate the whole skill of baseball batting, as a recently released virtual reality baseball training app does (Turner, 2015).

In large part because baseball batting performance in competitive leagues is represented by an array of statistics, researchers have been able to measure effects of pitch recognition training on performance. For example, the *Axon Sports* pitch recognition training application was made available to an NCAA Division-I college baseball team for self-directed use by players during the 2013 baseball season (Belling and Ward, 2015). Effects of the training program were measured by comparing the cooperating team's batting statistics in the season previous to using the *Axon Sports* system with statistics from the 2013 season. Use of the computer software was not guided or tracked by researchers, but was determined to have been effective because of statistically significant increases in the team's home runs, runs scored, and slugging percentage.

Fadde (2006) also demonstrated effects on batting performance associated with video-occlusion training of pitch recognition by comparing the batting performance of a group of players who received training with a control group of players on the cooperating team who did not receive video-occlusion training. Treatment and control groups were compared by ranking batters on the statistics of Batting Average, On-base Percentage, and Slugging Percentage. Using the Mann–Whitney *U*-test, batters in the treatment group ranked higher on all three batting statistics and significantly higher on Batting Average (Fadde, 2006). Despite the demonstrated effects of pitch recognition training on batting performance, however, these methods have yet to be widely adopted by teams as a routine part of preparing high-performance batters (Belling and Ward, 2015).

Not only does limited application of proven perceptual-cognitive training methods limit potential improvement of high-performance athletes but it also limits the potential that many expertise researchers envision for applying the theories, findings, and methods of sports expertise research to the training of macrocognitive skills in domains such as military and law enforcement (Eccles et al., 2008; Ward et al., 2008). Although years of controlled experimental studies have evidenced the expert performance approach (Abernethy, 1999; Williams and Grant, 1999) the expert performance approach is mostly likely to be adopted for training when it meets the instructional design challenge of fitting into existing workplace routines.

### Challenges for instructional designers who are designing training

When working on macrocognitive skill training, instructional designers need to balance research, training needs, and workplace constraints as they structure training curricula that aim to improve performance skills in the workplace (Richey et al., 2011). There are at least four challenges for an embedded expertise training program:

#### Instructional design challenge # 1: duration

What training duration is needed to make a meaningful difference in performance? Since instructional designers prize efficiency, duration is of considerable importance, as are timing and frequency of training events. Most of the perceptual-cognitive training studies reported in sport science literature were experimental training programs of limited duration, often with novice or intermediate trainees. These studies have served to validate perceptual training techniques and technologies but there is no indication that they have been sustained beyond the experimental context. Ideally, training for advanced performers in the workplace should be available when it is needed and individualized to address gaps between desired and delivered performance (Richey et al., 2011).

#### Instructional design challenge # 2: curriculum

Does the training program target specific macrocognitive skills associated with expert performance? Are there existing expert-novice academic studies that suggest target skills? If not, is it worth conducting a small-scale study to discover or confirm macrocognitive skills that differentiate known expert performers from less skilled performers, as Blair (2015) did to inform her design of a training program for peer academic coaches? Once target skills are identified then training tasks can be derived from or inspired by the representative tasks used in expert-novice research. Typically representative tasks focus on situation awareness or pattern recognition and involve: (1) Recall, (2) Detection, (3) Categorization, or (4) Prediction (Chi, 2006).

#### Instructional design challenge # 3: resource optimization

Can the program be implemented with limited resources? In part because of relatively limited budgets sport expertise researchers have developed approaches such as video-occlusion, which offers high functional fidelity but low psychological fidelity (it doesn't feel real) by decoupling perception and action for efficient and budget-friendly part-task training. Key concerns are if, when and how performers can recouple the perception-action link for transfer from the part-task training to whole-task performance (Farrow, 2013). *In situ* tasks that researchers have devised to measure learning gains can be repurposed as training tasks that enhance ecological validity. A training program implemented with competing athletic teams or other working professionals could include both highly targeted and efficient video-occlusion tasks and also transfer-appropriate *in situ* tasks.

#### Instructional design challenge # 4: buy in

Does the program have commitment from the on-the-ground personnel who influence the effort of trainees? Long-term sustainability often is tied to the initial buy. In sports training access to high-performance athletes, even when researchers are able to attain it (e.g., Hopwood et al., 2011; Mann et al., 2013), is not enough to ensure success. The attitudes of coaches or superiors toward a training program impact how trainees approach its implementation. Buy in cascades through the curricular design. Because baseball is very routinized in its approach to when and where athletes practice certain skills, a training curriculum has to strive to weave its activities into pre-established routines. Minimizing disruption of habits maximizes the chances of true buy in.

## Methods

### Training methods: case study of training baseball pitch recognition

The baseball training project reported here embodies the XBT approach that applies, but also modifies, techniques and technologies of expertise research in order to train key perceptual-cognitive skills and thereby accelerate expertise in already skilled performers. This case study with an NCAA Division-I college baseball team in the U. S. would be labeled a holistic design by Yin (2014); Campbell and Stanley (1963) would call it a one-shot case study. The training-based case study was conducted over a 10-month period in 2013–2014. The goal of the project was to create a training program that was based on research but also fit into established practice routines of the cooperating team.

#### Baseball context: the pitcher-batter matchup

For readers who may not be familiar with baseball a primer is provided (see Appendix) that provides some basic context. The central action of the game, sometimes called the game within the game, is the individual matchup of pitcher and batter. The act of hitting a round baseball with a round bat, which Ted Williams famously called “the single most difficult thing to do in sport” (Williams and Underwood, 1971, p. 3), affords batters very little margin for error in striking a pitched ball squarely and not popping up or grounding out because of off-centered contact.

At high levels of competition, with many pitchers throwing the ball over 90 miles per hour, batters have less than one-half second from release of the pitch until its arrival in the hitting zone (Bahill and LaRitz, 1984). Most batters take about 250 ms to swing a bat, leaving less than 250 ms (literally the blink of an eye) to decide whether to swing at a pitch and, if so, where to direct the swing. Batters can make fine mid-swing adjustments in the timing and direction of their swing, but only within a limited temporal and spatial window. Therefore, a batter's ability to perceive cues—whether consciously or not—from the pitcher's motion, the release of the pitch, and early ball flight can afford batter precious milliseconds of decision time.

#### Buy in: initiating the pitch recognition training program

The relationships of the cooperating teams' coaches with each other as well as with the players were central to implementing an innovative training program. The coaching staff (head coach, hitting coach, pitching coach, and volunteer assistant coach) was entering a second season with the team in 2014. Before the 2013 season the head coach, who had played for the same college and also played several years of minor league baseball, was hired to replace the previous coach. The head coach hired assistant coaches, who started as an intact staff in 2013. The first season with the team consisted of establishing expectations, policies, and procedures. The team had modest success in 2013, finishing in sixth place in their 11-team conference and thereby being the last team eligible for conference's post-season tournament.

After the initial season's experience, the hitting coach felt empowered to express his opinion that the team's top priority preparing for the 2014 season was to improve batters' pitch recognition. The head coach accepted the hitting coach's arguments that improved pitch recognition would lead to better plate discipline (batters refraining from swinging at pitches out of the strike zone), which—in theory—would reduce strike outs, increase bases-on-balls and on-base percentage (a combination of walks and hits), and runs scored per game. The head coach gave the hitting coach authority (although no budget) to design and install a pitch recognition training program. The hitting coach contacted the researcher and asked for help designing an extensive pitch recognition training program. The coach and the researcher undertook the project understanding that it would be developed iteratively since pitch recognition training studies (Burroughs, 1984; Fadde, 2006; Belling and Ward, 2015) used for guidance were limited in duration and integration.

The pitch recognition training program was initiated in September of 2013. All 18 position players (non-pitchers) provided informed consent and volunteered to participate in the pitch recognition training program. At the team's season orientation meeting the researcher gave a presentation on the sport science research behind the occlusion method of training pitch recognition. The head coach affirmed his support of the program and the hitting coach handed out a Hitting Manual that he had written and printed, which included descriptions of the pitch recognition drills.

#### Pitch recognition curriculum

Embedded training programs, in comparison to limited duration experimental training programs, need to have a guiding curriculum. While several sport science studies involved fairly sophisticated experimental training programs that included video-occlusion (e.g., Fadde, 2006; Hopwood et al., 2011) they were still limited duration experimental programs. The best example of a curriculum approach was a visual skills program conducted with a college baseball team over the course of 3 years (Clark et al., 2012). The program had distinct pre-season and in-season phases that included several different visual skills techniques and technologies, such as Nike Strobe goggles and Dynavision hand-eye reaction trainer.

For the pitch recognition training program reported here, the hitting coach and the researcher negotiated two key principles: (1) apply the relevant sport science with as much fidelity as reasonably possible, and (2) integrate pitch recognition training into established team practice routines. The later was important for sustainability of the training approach and was also necessary because of rules enforced by the National Collegiate Athletic Association (NCAA)—the ruling body of U. S. college sports—that restrict the number of direct contact hours per week between coaches and players.

The pitch recognition training program had several phases that made up a curriculum plan:

1. Fall Practice (mid-August through late October). Players worked with coaches in groups of four for 1 h 2 days a week. The hitting coach worked with players in the outdoor batting cage (a netted area approximately 20 feet by 60 feet) or the indoor batting cage depending on weather conditions. The session consisted of part-task batting drills, some of which were adapted to include elements of pitch recognition.
2. Winter Workouts (late October through December). Players self-monitored weight room workouts during a non-contact period with coaches, as defined by NCAA regulations. During the non-contact period a laptop computer with a prototype version of the *Axon Sports* computer application was set up in the baseball office and players used it voluntarily. The laptop was available in November and December.
3. Spring Practice (January through late February). The full team worked with coaches at the practice field, which included batting cage and bullpen area where pitchers practiced pitching to catchers at full pitching distance. Batters did familiar batting drills that had been introduced in fall practice and Bullpen Stand-In drill was added to practice session.
4. In Season (late February through May). During the season formal practice sessions were limited but some batters chose to incorporate batting cage drills and Bullpen Stand-In into their pre-game preparation. As with weight lifting, the established routine was heavy work during the off season and light maintenance work during the season, which typically included three games per week with travel to around half of the games.

While there have been several pitch recognition training interventions with college baseball teams they either focused on a short time frame (Burroughs, 1984; Fadde, 2006) or made a training technology available to a team for a full season but did not specify instructional activities (Belling and Ward, 2015). This was the first pitch recognition training program that featured a curriculum throughout the in-season and off-season phases of a sports year.

#### Computer-based pitch recognition component

**Figure 3:**
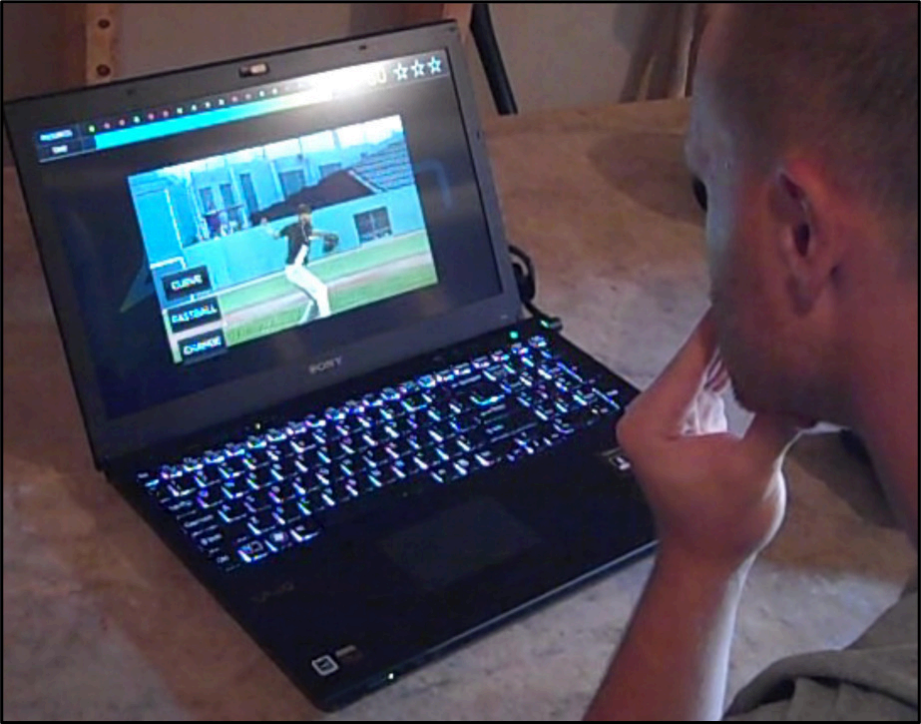
**Video-Simulation (courtesy *Axon Sports*)**.

*Axon Sports* provided the cooperating team with a prototype version of their pitch recognition application that ran on a 17-inch touch screen laptop computer (see Figure 3). The computer was available in the baseball office for voluntary and self-directed use by players. A player using the computer system would log in and then use menus to build a drill. The player selected:

1. Pitcher, from three pitchers that had different repertoires of pitches.
2. Batting side, either left-handed or right-handed batter viewpoints.
3. Drill type, including Pitch Type, Pitch Location, and Zone Hitting.

Players could return to in-progress drills at later sessions. The level of difficulty, which was determined by the amount of ball flight before occlusion, always started at the easiest level and advanced to more difficult levels as players achieved mastery scores, essentially beating the level in video game fashion.

Each round of a drill presented 20 pitches selected from a larger item pool. Players input multiple-choice answers (e.g., Fastball/Curveball/Changeup) by pressing a button on the touch screen. The computer program accepted the player's input, judged correctness of the input, displayed the correct answer, and played an audio tone to indicate correct or incorrect input. The program automatically played the next video pitch and presented a score at the end of the drill. Most drills took about 5 min to complete. At the end of drill the computer would automatically progress the player the next level of the drill if the player had reached criterion score. Although players usage was not tracked, 14 out of 18 players reported that they used the *Axon Sports* computer application at least once and 10 of the players reported that they reached the highest level of progressive difficulty in several video drills.

#### Design and implementation of in situ batting cage drills

The researcher worked with the hitting coach to overlay a pitch recognition element onto several routine batting cage drills that players did during small group workouts. A key challenge was to devise live visual occlusion tasks. Many sport science studies have used liquid crystal occlusion glasses for *in situ* occlusion tasks. Occlusion glasses instantly change from clear to opaque when sent an electronic signal, effectively cutting off the wearer's vision.

Several studies have used occlusion glasses in cricket and baseball batting tasks (e.g., Müller and Abernethy, 2006; Müller et al., 2010, 2015b). In these *in situ* tasks, batters faced a live baseball pitcher or cricket bowler. In some studies, batters were directed to swing at the pitched ball, even after their vision had been occluded. Researchers gained at least two benefits from *in situ* batting tasks with occlusion glasses. According to ecological dynamics theory (Davids et al., 2013) a batter producing the realistic motor action of swinging his bat should engage the appropriate dorsal stream and maintain the perception-action link. In addition, some studies paired *in situ* occlusion with chronometric analysis using high-speed video cameras and force plates to ascertain precisely when and how a cricket or baseball batter synchs his swing to the movements of the pitcher (Müller et al., 2009, 2014).

While delivering substantial research benefits, however, there are many issues involved with live occlusion tasks. It can take up to 2 h to conduct a test on each subject, which may be tolerated for a one-time experiment but not for routine practice sessions. There is also the possibility of cricket or baseball batters being hit by a pitch when their vision is occluded. Although injury potential can be lessened by using low-impact balls and outfitting batters with elbow guards, the chances of getting hit by a pitched or bowled ball are much higher in training situations than in testing situations because many more pitches are faced in less controlled contexts.

**Figure 4:**
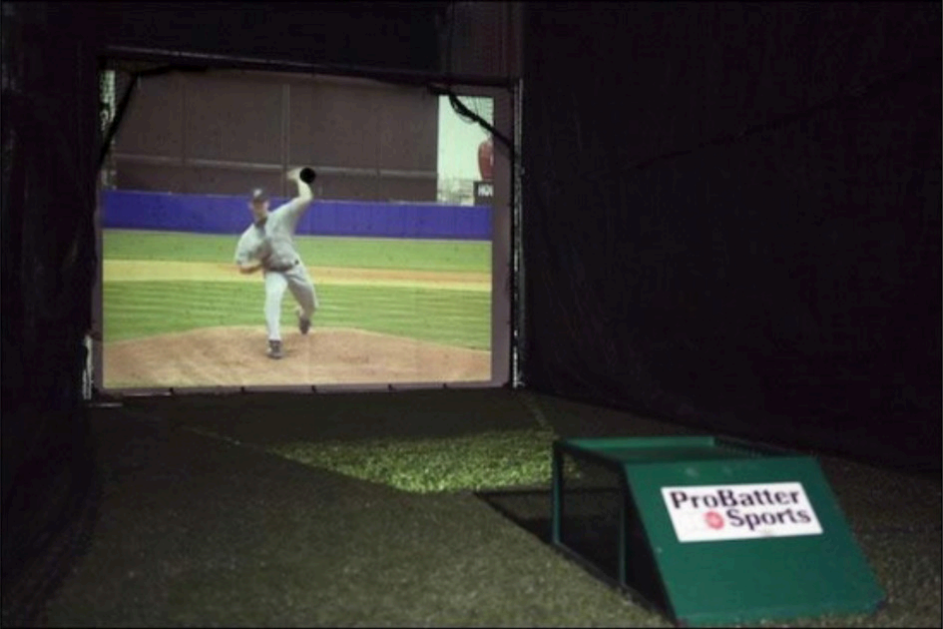
**Video pitching machine (courtesy ProBatter)**.

Using live pitchers for *in situ* testing is problematic because the same pitchers can't pitch to every batter. Müller et al. (2015a) argue that the skill of pitch recognition is assumed to generalize across numerous pitchers, so variety is desirable. While certainly a legitimate point for training, testing of pitch recognition that will be used to compare players should certainly be tested against consistent pitchers. The problem can be lessened by using a video pitching/bowling machine, such as *ProBatter*, which displays a video image of a pitcher or bowler that matches the type of ball being delivered (see Figure 4). However, the $40-50,000 price of professional grade *ProBatter* is out of the range of most teams.

**Figure 5:**
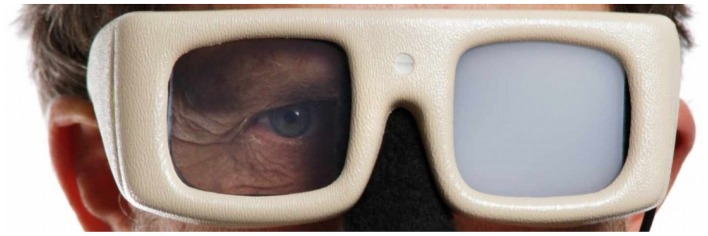
**Occlusion Glasses (courtesy Translucent Technologies)**.

**Figure 6:**
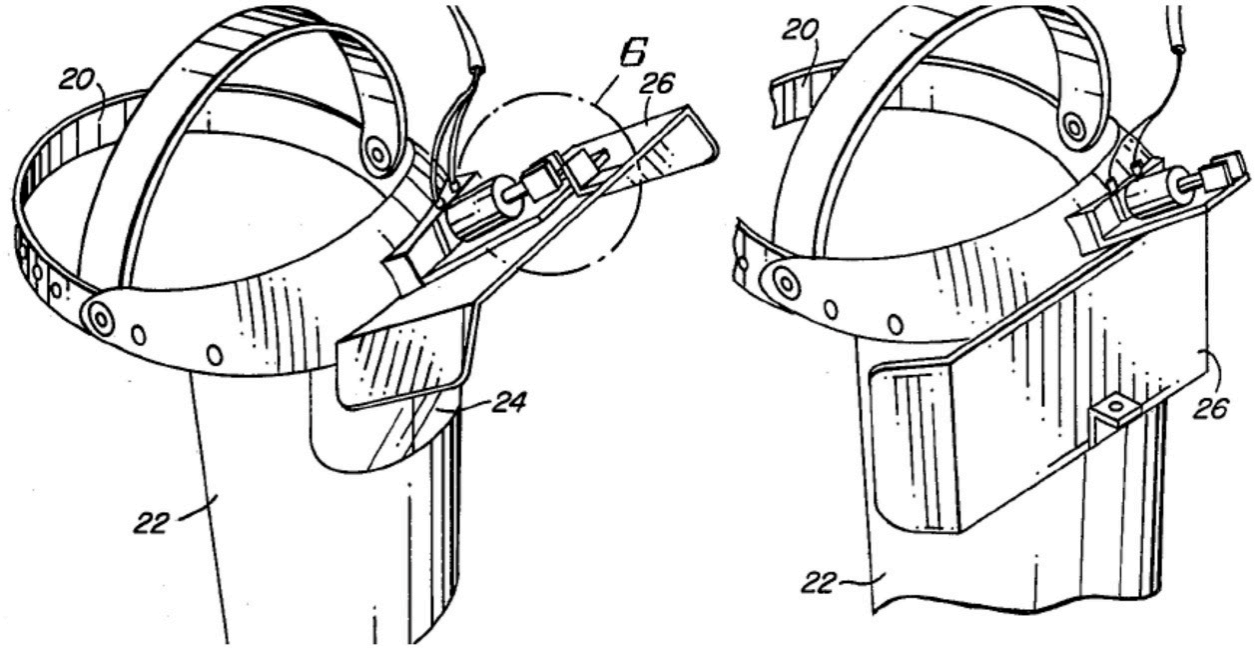
**Visual Interruption System patent illustration**.

Figure 5 shows a pair of liquid crystal display glasses and Figure 6 shows the patent drawing of a novel device that Burroughs (1984) invented to test and train pitch recognition. The Visual Interruption Systems featured a batting helmet equipped with a plate that would drop in front of a batter's eyes to occlude his vision. The V.I.S. system was triggered by a batter's weight shift while stepping on a force plate.

**Figure 7:**
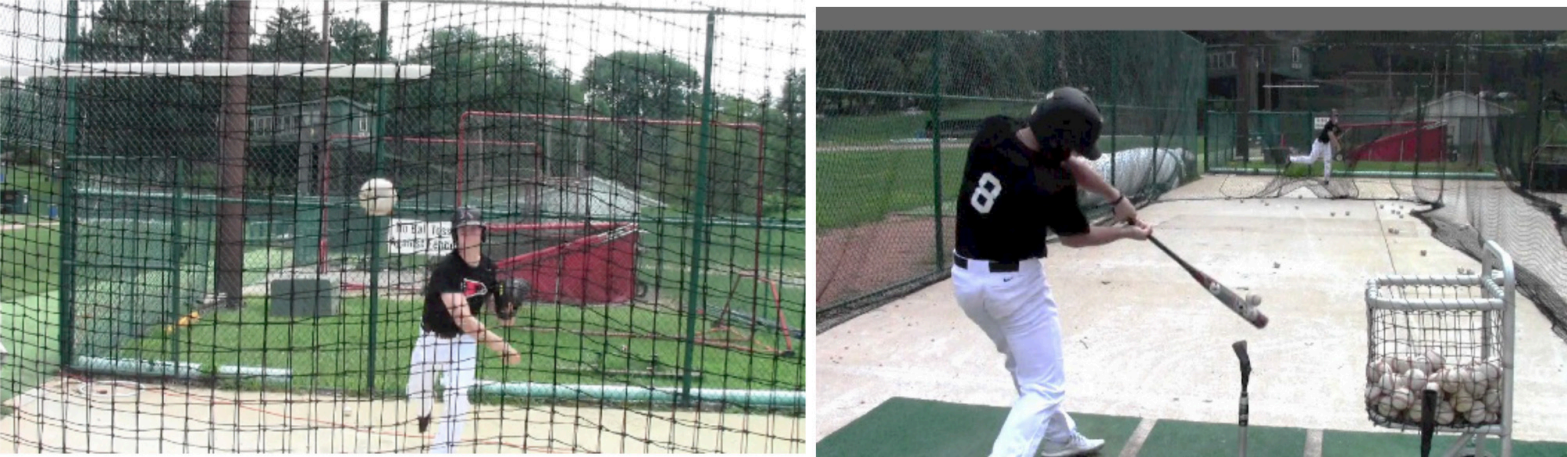
**Net Occlusion Drill (https://www.youtube.com/watch?v=ZaXYlcw1uM0)**.

While occlusion glasses are a valued tools in research settings they may be too complex, expensive, and intrusive to be used in training settings. However, training goals do not require the strict occlusion variations that testing and research goals require. The hitting coach and researcher developed an *in situ* occlusion task that did not require technology but maintained the operational principles (Gibbons, 2009) of occlusion. As shown in Figure 7, *Net Occlusion Drill* involved one player standing behind a net drawn across the batting cage and throwing a simulated pitch into the net, effectively occluding ball flight. The player throwing the simulated pitch (usually another batter rather than a real pitcher) showed authentic pitch release cues, such as the skinny wrist many pitchers show when throwing a curveball. The batter read pitch release cues and called the type of pitch aloud. Depending on the objective of the drill (e.g., “hit fastballs”) the batter could strengthen the association of recognizing the pitch type and hitting a ball off of the tee.

Net Occlusion Drill has several advantages for the team over batting practice facing a live pitcher. One is that a non-pitcher can throw the stimulus pitches so that pitchers are not being stressed by pitching to batters. Another advantage is that the part-task objective of recognizing pitch types does not become conflated with the full task of hitting the pitch. Net Occlusion Drill represents the second of three levels of video-simulation fidelity proposed by Müller et al. (2015a):

1. Video Simulation with Non-Motor Response,
2. Video Simulation or Virtual Reality with Motor Response,
3. *In-Situ* with Motor Response

#### *In situ* in the bullpen: attention occlusion

**Figure 8:**
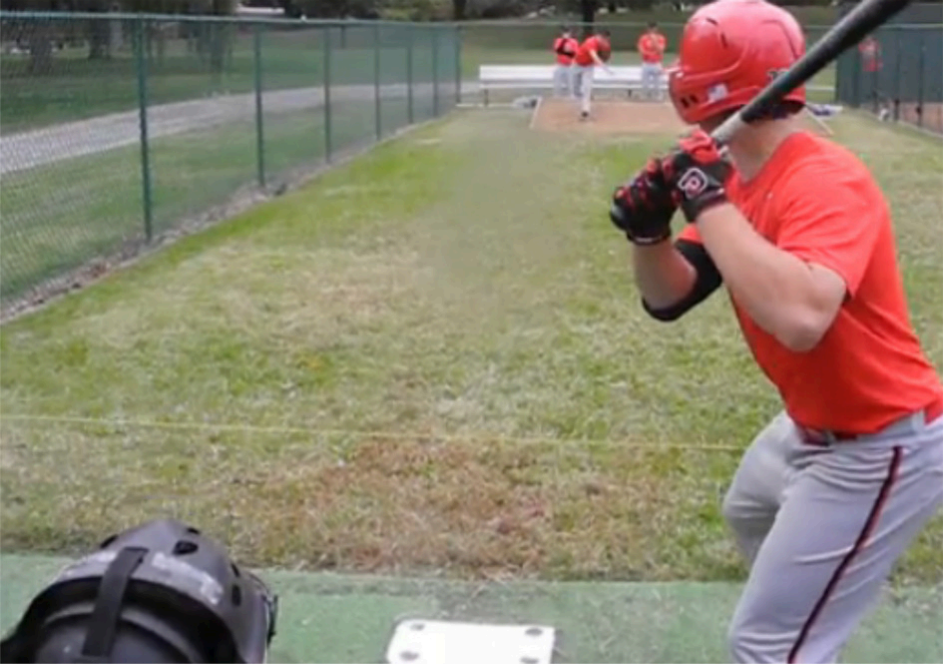
**Bullpen Stand-In Pitch Recognition Drill**.

Another live occlusion drill developed for the pitch recognition training program simulated computer video-occlusion by “standing in” while the team's pitchers were practicing pitching in the bullpen (a designated area at baseball fields where pitchers practice or warm up for a game). A batter would assume his normal position in the batter's box but would not swing his bat (see Figure 8). Instead, the batter would call aloud the type of the pitch being delivered before the pitch hit the catcher's mitt. *Bullpen Stand-In Drill* was developed with input from a batting coach who uses it with minor league batters in his major league baseball organization (White, 2014).

Stand-In is a routine practice activity that players have been doing for many years and that is usually associated with tracking pitches from the pitcher's hand to the strike zone. Bullpen Stand-In Drill changes the batter's focus to identifying cues in the pitcher's windup, release of the pitch, and early ball flight. In a video-occlusion context, occlusion removes tracking of pitches by cutting to black during ball flight. In the bullpen, the batter shifts his attention from visual pattern recognition as a System1 cognitive process (Kahneman, 2011) to verbal message construction in System 2, thereby cutting off his attention. Calling out pitch type before the pitch hits the catcher's mitt forces the *attention occlusion* into the time frame from pitch release through 1/3rd ball of flight that expert-novice research found to be the window of maximum expert advantage (Paull and Glencross, 1997). While the cognitive process of attention occlusion is speculative at this point, calling the pitch before it hits the catcher's mitt appears to effectively occlude batters' attention in the critical pitch recognition window.

To be consistent with the computer-based occlusion drills, batters would choose call aloud the pitch type (e.g., Fastball, Curve, or Changeup) or location (ball or strike). Attention occlusion is a level one simulation (Müller et al., 2015a) in that the players' response is verbal rather than a relevant motor movement. When players had been doing Bullpen Stand-In Drill for a couple of weeks the coach gave them a ghost bat that he'd created by sawing off a broken metal bat to about one-foot in length and adding weight to make it feel more like a real bat. The shortened bat meant that the batter could swing at a pitch but without making contact with the ball since bullpens are not designed for batting practice. Allowing batters to swing the ghost bat was satisfying to players and arguably increased the ecological validity (Bootsma and Harvey, 1997) of the Stand-In drill. With the addition of the ghost bat, Bullpen Stand-In Pitch Drill became a level-two simulation in which batters input their pitch recognition verbally and input their swing decision with an authentic movement.

Several of the players were initially reluctant to call pitches out loud, perhaps because it made their mistakes public. The coach countered by reminding players that, “If you're getting them all right, you're doing it all wrong.” He wanted players leaving their comfort zone to call pitches earlier. Bullpen Stand-In Drill needed to be carefully monitored to have the desired cognitive training effect. When executed properly, though, it captured value of *in situ* training while addressing several issues associated with occlusion glasses. It did not require expensive or complex technology and it took advantage of real pitchers without adding to their pitching load.

### Research methods: procedures used in the study

Participants in the training program included all 18 of the position players on the cooperating team. The mean age of the participants was 20.7 years. All participants were white males. The participants had been on the cooperating team's roster for an average of 2.5 years at the start of the project. All of the batters who volunteered to participate in the pitch recognition training program received training, so no internal control group of untrained batters was designated as done in previous studies of pitch recognition training (Fadde, 2006). The unit of analysis was batting performance of the team as a group.

#### Batting performance

The primary research question was whether the embedded pitch recognition training program would lead to improvements in team batting performance. The independent variable was the pitch recognition program in its entirety, including the computer-based video-occlusion application and the *in situ* pitch recognition drills. There were two dependent variables, both based on season team batting statistics published by the NCAA (2015). The first DV was the batting performance of the cooperating team in baseline (2013), implementation (2014), and adoption (2015) seasons. Serving as control, batting statistics were compared to the mean values on the same statistics of all the teams in the cooperating team's athletic conference. The second DV was change in the cooperating team's batting statistics from baseline season (2013) to implementation season (2014) seasons.

Analysis of the change in the team's batting performance was compared to change in batting performance over the same seasons by a comparable team in the same athletic conference. The team designated as the comparison team was the conference team most similar to the cooperating team. Both the cooperating team and the comparison team returned 7 out of 8 batters from their 2013 starting lineups for the 2014 season. Both teams made the 6-team post-season tournament in both the 2013 and 2014 seasons. Both teams improved their win-loss record and position in the conference standings from 2013 to 2014, with the cooperating team winning the 2014 conference regular season championship and the comparison team winning the conference's 2014 post-season tournament. Comparing the cooperating team's change in performance to a selected and comparable conference team, rather than using the mean performance of the whole conference, let the research address the coaches' question of whether any improved performance was “beyond what would be expected from a good team getting better.”

The batting statistics analyzed were the team performance measure of Runs-per-Game along with the individual performance measures of Batting Average, On-base percentage (which includes walks and hits), and Slugging Percentage (which counts all bases and is considered to be a measure of power hitting)—three statistics that thought to provide a rounded profile of batting performance (Weinberg, 2014). Other statistics analyzed included Walk Rate, Strikeout Rate, and Walk-to-Strikeout Ratio that are considered to represent plate discipline (Panas, 2010). Scoring (Runs-per-Game) is the most basic measure of team offensive performance; Walk-to-Strikeout Ratio is the most basic measure of individual plate discipline.

#### Pitch recognition testing

As noted earlier, testing and training have a close relationship in expertise-based training. The pitch recognition training project described here offered several opportunities to test not only for group differences, as has been done in the expert-novice research paradigm, but also test for individual differences and individual development as sport science researchers are just beginning to pursue. As a training project, however, ideal testing conditions for research purposes were sometimes compromised for the sake of team preparation and competition.

After the pitch recognition training program was underway, a validated video-occlusion Pitch Recognition (PR) test became available and was administered to batters on the cooperating team. Later, a second video-occlusion pitch recognition test became available and was also administered to the cooperating team. Both PR video tests showed pitches from a perspective closely, but not exactly, depicting the view of a participating batter. However, the tests differed in the occlusion points that were used. While the seminal laboratory-based expert-novice study of pitch recognition (Paull and Glencross, 1997) used an array of occlusion points cutting off pitches before, at, and after the pitcher released the pitch, testing professional baseball batters in the field required researchers to construct shorter video-occlusion tests.

The first video occlusion test developed for testing professional players, heretofore called the Pre-Release Test, used video clips of pitches that were occluded at Release of the pitch and at two occlusion points before Release. The Pre-Release video-occlusion test was formally validated and used to test professional players competing in the Australian Baseball League (Moore and Müller, 2014) and later used to test minor league players in the United States (Müller and Fadde, 2016). The second test, heretofore called the Post-Release Test, was developed later and featured pitches that were occluded at Release and at two occlusion points after the pitcher released the pitch. Both the Pre-Release and Post-Release tests were administered to batters on the cooperating team, which allowed several questions about pitch recognition testing to be addressed:

1. Would either or both PR tests differentiate groups of batters by skill level?
2. Would either or both PR test correlate with batting performance?
3. Would the Pre-Release and Post-Release tests correlate with each other?
4. What insights might be gained from PR testing for coaching purposes?

The Pre-Release PR test was administered in the fall of 2014. The 2014 baseball season finished in May and the test was administered at the beginning of the next school year (2014–2015), which is considered to be part of the 2015 season. The college baseball season is split into a fall period with organized practice and the competition portion of the season in the spring of the next calendar year. Of 20 players who took the PR test in Fall 2014, 10 played regularly (100+ Plate Appearances) in the 2014 season or would be regular players in the 2015 season. The other 10 players played part-time. The PR scores of these two groups were compared in an adaptation of expert-novice methodology. Batters' individual scores on the Pre-Release PR test were also correlated with season batting statistics of seven batters who had been regular starting players in the 2014 season.

The Post-Release PR test was administered twice in the fall of 2015, about 6 weeks apart. Scores on the first Post-Release PR test were correlated with scores on the Pre-Release PR test. The two administrations of the Post-Release PR test were correlated with each other to address the question of whether pitch recognition is a stable trait of batters or a fluctuating state. Since regular and extensive testing of the pitch recognition skill of batters in many contexts will require different PR tests it is important to develop methods of validating new tests. The coaches of the cooperating team embraced testing for development of their players as well as advancing the science of pitch recognition testing.

## Results

### Team batting performance

Table 1 shows the cooperating team's batting statistics for the 2013 season (baseline), the 2014 season (implementation), and the 2015 season (adoption). The mean batting statistics of all the teams in the cooperating team's athletic conference serve as control. Change in the statistics of *Runs-per-Game* and *Walk-to-Strikeout Ratio* (BB/K) are bolded in Tables 1, 2 because these are the most relevant statistical representations of team offense and individual plate discipline, which is defined as swinging at pitches that are in the strike zone and refraining from swinging at pitches that are out of the strike zone. Values that are shown in parentheses in the Differences columns indicate lower performance by the cooperating team. Strikeouts are reverse scored; a lower number is considered to be a better performance.

**Table 1:** **Differences in Batting Statistics: Cooperating Team vs. Conference**.

	**2013**			**2014**			**2015**		
	**Team**	**Conf**.	**Diff**.	**Team**	**Conf**.	**Diff**.	**Team**	**Conf**.	**Diff**.
Runs Per Game	5.8	6.2	**(6%)**	8.6	6.3	**37%**	9.4	7.2	**17%**
Batting Average	0.286	0.291	(2%)	0.326	0.290	12%	0.324	0.301	8%
On-base Pct.	0.372	0.371	–	0.407	0.375	11%	0.419	0.385	9%
Slugging Pct.	0.390	0.418	(7%)	0.468	0.422	11%	0.519	0.435	19%
Home Runs	11	21	(48%)	25	23	9%	39	35	11%
Base-on-Balls	108	111	(3%)	140	120	17%	171	126	36%
Strikeouts (K)	217	200	(9%)	182	199	9%	219	203	(8%)
BB/K Ratio	0.50	0.56	**(11%)**	0.77	0.60	**28%**	0.78	0.62	**26%**

*Change in key PR stats bolded*.

**Table 2:** **Changes in Batting Statistics: Cooperating Team vs. Comparison Team**.

	**Cooperating Team** **2013–2014 Change**			**Comparison Team** **2013–2014 Change**		
Runs Per Game	5.8	8.6	**48%**	6.6	6.8	**3%**
Batting Average	0.286	0.326	14%	0.290	0.304	5%
On-base Pct.	0.372	0.407	9%	0.372	0.383	3%
Slugging Pct.	0.390	0.468	20%	0.413	0.464	12%
Home Runs	11	25	127%	17	27	59%
Base-on-Balls	108	140	30%	127	124	(2%)
Strikeouts (K)	217	182	16%	189	200	(6%)
BB/K Ratio	0.50	0.77	**54%**	0.67	0.62	**(7%)**

*Change in key PR stats bolded*.

In the 2013 season, the cooperating team was below conference mean on almost all batting statistics. In the 2014 season, which included pitch recognition training, the cooperating team was higher than conference means on all of the analyzed batting statistics. In 2015 the cooperating team's batting statistics were again consistently better than the mean scores of the conference, with the exception of strikeouts. As context in interpreting batting statistics, general benchmarks at the major league level include: 0.300 for Batting Average, 0.375 for On-base Percentage, 0.450 for Slugging Percentage, and 0.500 for Walk-to-Strikeout Ratio (BB/K).

While Table 1 shows clearly superior batting performance in the implementation year (2014) following pitch recognition training, the central question of whether pitch recognition training was associated with improvement in batting performance from baseline to implementation seasons was evaluated by comparison with improved performance of a similarly successful team (see Table 2).

An effect size for improvement in the key batting statistic of Walk-to-Strikeout Ratio (BB/K) from 2013 to 2014 was calculated using the mean BB/K of 12 batters on the 2013 roster (mean = 0.51; *sd* = 0.22) and 11 batters, including six hold-overs from 2013, on the 2014 roster (mean = 0.80; *sd* = 0.37) who had a minimum of 50 plate appearances (as Belling and Ward, 2015). The effect size was large (*d* = 0.953) and significant (*p* = 0.017) at *p* < 0.05.

**Figure 9:**
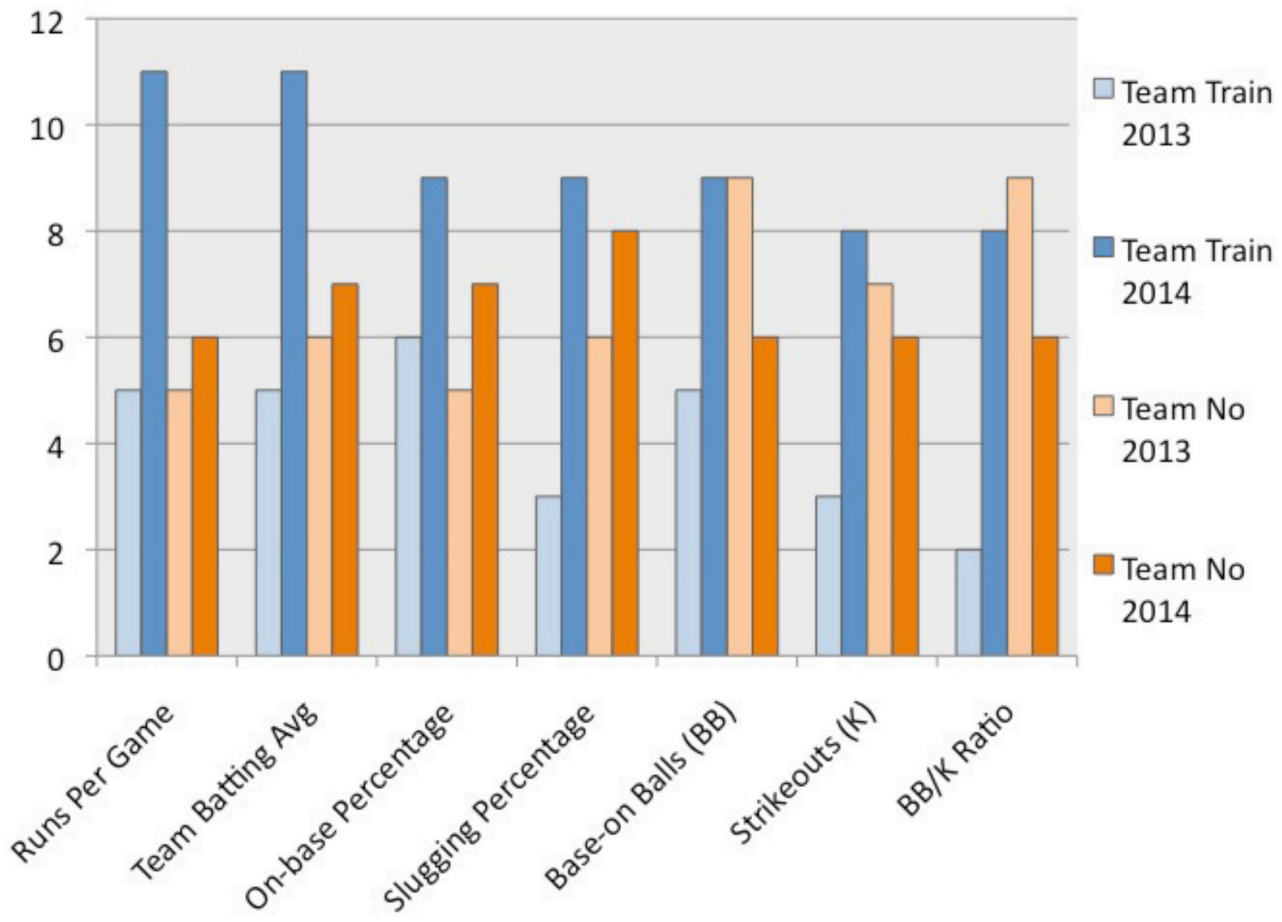
**Ranking of Training and No Training Teams in Conference(11 = best; 1 = worst)**.

While coaches were satisfied with percentage of change as evidence of improvement, as shown in Tables 1, 2, the research question of whether pitch recognition training was associated with improved overall batting performance required determining the statistical significance of overall performance improvement from 2013 to 2014. Overall season-to-season improvement was assessed by comparing the conference ranks on selected batting statistics in 2013 and 2014 of both the cooperating (training) and the comparison (no training) team (see Figure 9). Mann–Whitney *U*-test of rank correlation, scaled for small *n*, was used to compare 2013 and 2014 seasons as a whole for each team. With 11 teams competing in the conference, the top rank score was “11” and “1” was the bottom rank score. Applying a one-tailed analysis with alpha of *p* < 0.05, the cooperating team's overall ranking on the selected batting statistics was significantly higher (*p* = 0.0005) in 2014 than in the 2013 season. The same analysis conducted on the comparison (no training) team's improvement from 2013 to 2014 was not significant (*p* = 0.4364). Figure 9 graphically displays the cooperating team improvement “beyond expectations of a good team getting better.”

After implementation of the pitch recognition training program for the 2014 season, the cooperating team continued to incorporate pitch recognition training, even without the *Axon Sports* computer application or direct involvement of the researcher. Although not as dramatic as the improvement from the baseline season (2013) to the implementation season (2014), the cooperating team continued to improve in the adoption season of 2015 (see Table 3).

**Table 3:** **Change in Batting Statistics for Cooperating Team**.

	**2013–2014 Change**			**2014–2015 Change**		
Runs Per Game	5.8	8.6	48%	8.6	9.4	9%
Batting Average	0.286	0.326	14%	0.326	0.324	<1%
On-base Pct.	0.372	0.407	9%	0.407	0.419	3%
Slugging Pct.	0.390	0.468	20%	0.468	0.519	11%
Home Runs	11	25	127%	25	39	56%
Base-on-Balls	108	140	30%	140	171	22%
Strikeouts (K)	217	182	16%	182	219	(20%)
BB/K Ratio	0.50	0.77	54%	0.77	0.78	1%

Of particular note in the 2015 season was the increase in home runs while also increasing in strikeouts, which reflected the hitting coach's 2015 hitting theme of being more aggressive at the plate. While batters struck out more, they also walked more, hit more home runs, and scored more runs. The 2015 batting statistics counter the common concern of coaches that training pitch recognition may lead to overly selective, and therefore passive, batters.

### Pitch recognition testing

The pitch recognition training project offered numerous opportunities for testing the PR skills of batters on the cooperating team. Administering two different video-occlusion PR tests to batters on the cooperating team permitted several questions related to PR testing to be addressed.

1. Would either or both PR tests differentiate groups of batters by skill level?
  Batters on the cooperating team were tested using a validated video-occlusion Pre-Release PR Recognition test (Moore and Müller, 2014) in advance of the 2015 season. The expert-novice paradigm was adapted, as Moore and Müller (2014) did, to compare a group of higher-skilled batters (players who were regularly in the starting lineup in either 2014 or 2015 seasons) with a group of lesser-skilled batters (non-regulars). The higher-skilled group's mean PR score was 58.8 while the less-skilled group's mean PR score was 52.1; the difference (*p* = 0.1304) was non-significant at *p* < 0.05.
2. Would batters' PR test scores correlate with batting performance?
  The Pre-Release PR test scores of individual batters were correlated with the batters' 2014 season batting statistics for Batting Average, On-Base Percentage, Slugging Percentage, Walk Rate, Strikeout Rate and Walk-to-Strikeout ratio. Using a minimum participation rate of 100 Plate Appearances (Moore and Müller, 2014), 11 of 18 batters tested qualified for the analysis. Using Pearson product moment coefficient, no significant correlations were found (at *p* < 0.05), a finding that is consistent with a study of minor league batters that found a significant correlation only for Walk Rate at one pre-release occlusion point (Müller and Fadde, 2016). The Post-Release PR test scores of six players who had played in the 2015 season were analyzed but did not significantly correlate with any of the batting statistics, in part because of the small number of players. However, the Post-Release PR scores can potentially be correlated with the 2016 batting statistics that will be generated by up to 18 batters who took the at least one version of the Post-Release test in fall 2015.
3. Would the PR tests correlate with each other?
  Only six batters took both the Pre-Release video PR test (in Fall 2014) and the Post-Release video PR test (in Fall 2015). The correlation between batters' scores on the two tests was moderate to strong (*r* = 0.707) but not significant (*p* = 0.117). The finding suggests that one validated video PR test can potentially be used to validate a second video PR test, but with further investigation needed.
  The Post-Release PR test was administered twice in fall 2015 with 14 out of 18 batters on the cooperating team's roster completing both tests, which were given about 6 weeks apart. Mean PR score on the first Post-Release test was 62.7 and the mean score on the second administration of the Post-Release test was 61.2, producing a moderately strong correlation (*r* = 0.53) that approached significance (*p* = 0.052). The correlation suggests that taking the video test with no item or summary feedback leads to minimal, if any, learning effect. At least provisionally, either PR video test could be used as both a pre-test and a post-test for research or training purposes. It also suggests that the PR test measures a fairly stable trait. Whether and to what extent batters' pitch recognition skill can change as a result of training, experience, or maturation—and whether changes can be measured with a video PR test—remain to be investigated. Being able to use the same test for repeated measures can be an important tool for coaches as well as researchers in addressing these questions.
4. What insights might be gained from PR testing for coaching purposes?
  The Pre-Release PR score of batters on the cooperating team was 55.40 (*sd* = 11.12), with PR scores ranging from 33 to 75. By comparison, 34 minor league baseball players completing the same video-occlusion test scored an overall mean PR score of 60.25 (Müller and Fadde, 2016). As noted above, players' individual scores on the Pre-Release PR test did not correlate directly with any individual batting statistics. One reason for the lack of correlation between test scores and performance is that hitting is an exceptionally complex system of psychological, cognitive, perceptual, and psychomotor subskills. Even a highly valid test of any one component skill is unlikely to predict overall skill performance. However, an astute coach can use measurement data on any or all components to inform selection and development of players.

Two batters' scores on the Pre-Release PR test illustrate how the same PR test score can have different implications for different players. The batters both achieved a score of 75 on the Pre-Release test, the highest scores of the 18 players taking the test. One of the players was a senior backup catcher whose primary hitting attribute was a good eye, that is, the ability to predict which pitches would or would not be in the strike zone. However, limited athleticism and several injuries over his career had led to limited playing time other than pinch hitting (batting in place of another player). His high PR score was consistent with his value and role on the team.

The other player to score a 75 on the Pre-Release PR test illustrates a potential use of PR testing to inform coaches' decisions about playing time or training approaches. The batter had a 0.242 Batting Average (compared to mean team Batting Average of 0.306) as a semi-regular in the 2014 season, his sophomore season. He then enjoyed a breakout season in 2015 with a Batting Average of 0.318 (team mean BA = 0.324) and hit a team-leading 13 home runs. Although providing only anecdotal evidence, the coaches' decision to keep this player in the lineup despite relatively poor batting performance appears to have been affirmed when the player's physical maturity and batting technique caught up with his advanced batting eye. PR testing has considerable utility to coaches if it reveals or confirms that a player has perceptual-cognitive skills that may not effect his progression from competence-to-proficiency but may play a role in accelerating the player's progression to expertise (Dreyfus, 2004).

## Discussion

### Limitations and future research

The study focused on just one macrocognitive aspect of baseball batting, pitch recognition, at the expense of other macrocognitive skills such as option generation involved in anticipating types of pitches based on game situations and opponent tendencies (Gray, 2002; Lebiere et al., 2003). Future studies could incorporate what Ted Williams called “proper thinking” about pitch probabilities into pitch recognition training (Ward et al., 2013; Cañal-Bruland and Mann, 2015; Cañal-Bruland et al., 2015).

The validated Pre-Release and Post-Release pitch recognition tests were not yet available when the pitch recognition training project started, so systematic pre/post-testing of PR skills before and after the initial implementation season was not possible. Although the cooperating team plans to continue testing and training pitch recognition it is unlikely that an entire group of players will be tested and start a training program at the same time. When opportunities for embedded training with competing teams arise, researchers must balance the value of critical pre-implementation testing with the need to fit into a team's established routines.

Testing, both video-based (Belling et al., 2015) and *in situ*, should have a larger role in future training programs, in part to address important and largely unknown questions about the state-vs.-trait nature of macrocognitive skills such as pitch recognition. Are these skills stable traits or can they be improved through targeted training? How much training, and of what type, leads to the most improvement? What are the minimum levels of physical, cognitive, and technical development needed in order to benefit from expertise-based training? Would the training methods used with Division-I college baseball players also work with more advanced professional batters, or with high school or even younger batters? Hopefully, embedded training/research projects address these questions in the process of implementing authentic macrocognitive training programs in military and other time-restricted, high-stress performance domains (Fadde, 2010, 2012).

## Conclusions

The findings of this case study don't support generalizing results beyond the specific team, training program, and performance domain. However, the performance gains of the cooperating team were dramatic enough to invite other baseball teams to develop, implement, and assess at least a portion of the pitch recognition curriculum developed in this study. In addition, researchers and trainers working in other domains may consider developing, implementing, researching, and reporting training programs similarly focused on specific and known macrocognitive components of performance in a variety of high-performance jobs. In military contexts, for example, recognition-based tasks such as patrol leaders spotting roadside explosive devices or landing signal officers waving off a pilot may be amenable to accelerated expertise through expertise-based training.

XBT champions efficiency, even in the nebulous realm of expertise. By focusing on instructional methods rather than technology-driven delivery systems (Clark, 1983), by holding to operational principles of a learning and performance systems (Gibbons, 2009) rather than satisfying, but not always optimal, whole-task learning experiences we are more likely to avoid building the wrong simulation (Foshay, 2006). Instead, we can target identified macrocognitive subskills of expert performance using representative tasks that favor cognitive fidelity over physical fidelity (Fadde et al., 2007) and thereby accelerate expertise in systematic and affordable ways.

Reflecting on the framework of research in laboratory, field, and workplace settings (see Figure 1) the theories, findings, models, methods, and representative tasks that emerge from expertise research deserve to be more widely applied in embedded macrocognitive training programs. In many domains and workplaces much more important than baseball the instructional design, human factors, and expert performance communities need to more quickly get more performers over the bars of expertise and expert performance (Hoffman et al., 2014).

Embedded training programs are likely to have widely varied content, contexts, and fidelity of implementation, but if they focus on key operational principles derived from laboratory and field research settings then they can potentially advance both research-based practice and practice-based research. Ideally, the workarounds and modifications that inevitably emerge from embedded real-world training programs should feed questions back to the basic research community so that they can be thoroughly investigated in controlled laboratory and field research settings. An example is the *attention occlusion* method that was adapted to contextual constraints but should be validated in the laboratory, perhaps using EEG instrumentation to observe specific points in time where a literal spike of pitch recognition is observed (Houdé et al., 2000; Muraskin et al., 2013; Park et al., 2015).

Instructional design theorists and practitioners have important roles to play in collaborating with cognitive psychologists in the human factors and naturalistic decision making communities to develop approaches that train macrocognition in the workplace (Fadde and Klein, 2012). The theories, findings, and methods of expert performance research need to be translated into focused workplace training programs that meet the challenges of duration, curriculum development, resource optimization, and buy in from on-the-ground practitioners. In summary, expertise-based training that applies research methods such as temporal occlusion in the context of workplace training can provide efficient and effective methods of systematically training aspects of performance that are typically assumed to come only with innate talent or massed experience.

## Author contributions

The author confirms being the sole contributor of this work and approved it for publication.

## Funding

OpenSIUC, an institutional repository offering permanent, reliable, and free access to research and scholarly material produced at Southern Illinois University (http://opensiuc.lib.siu.edu/).

### Conflict of interest statement

The author declares that the research was conducted in the absence of any commercial or financial relationships that could be construed as a potential conflict of interest.

## Figures and Tables

**Figure A1:**
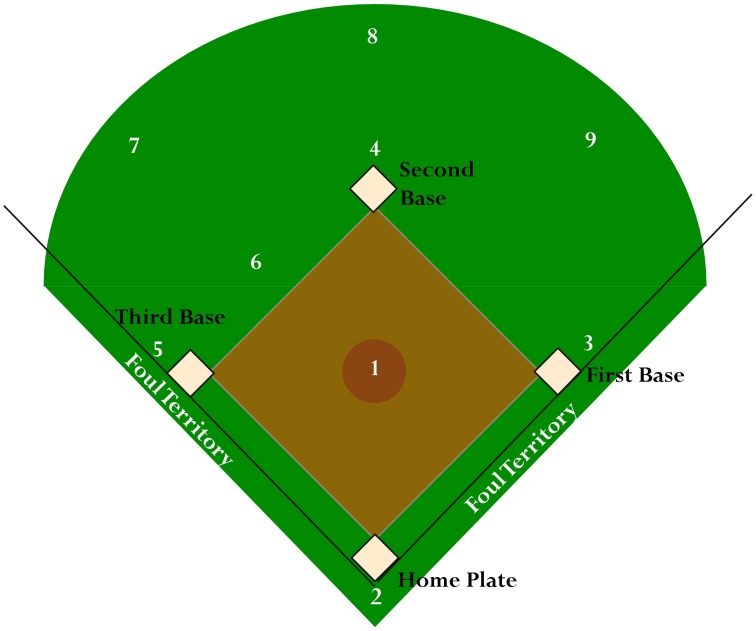
**Diagram of baseball diamond (courtesy:cliparthut.com/)**.

## Supplementary material

### Videos

1. “Doing Damage at the Plate by Training Pitch Recognition,” an informational clinic about the pitch recognition training program presented at the American Baseball Coaches Association conference, Orlando FL, January 2015: https://www.youtube.com/watch?v=ukuKbj_5sUQ
2. “Look, 'Ma, No Hands,” a presentation about applying sports expertise methods to training in military domains. Inter-service/Industry Technology, Simulation, and Education Conference (I/ITSEC), Orlando FL, December 2010: https://vimeo.com/36425360

## Appendix: baseball primer

A baseball game features nine players in each team's lineup. The teams alternate turns batting and playing the field for each of nine scheduled innings or until a winner is determined; there are no ties in baseball. A team can score runs (points) only when batting. As shown in Figure A1, the game is played on a field vaguely shaped like a diamond. Home plate and three bases define the infield, all 90 feet apart and separated by wide dirt running paths. When a team is in the field, the pitcher stands in the middle of the diamond (position 1 in Figure A1) and throws pitches to the catcher (position 2) who is positioned behind home plate, approximately 60 feet from the pitcher. Four infielders assume positions (3, 4, 5, and 6) loosely associated with each base. Three outfielders (positions 7, 8, and 9) patrol the expanse of grass between the infield and the outfield fence. The fence can be symmetrical or have odd shapes, such as legendary Fenway Park that was built to fit on a city block in Boston. The distance from home plate to the outfield fence varies and is not officially defined but is typically between 300 and 400 feet from home plate. When a pitched ball is batted into the fair playing area fielders can record an out by either catching the ball in the air or fielding the ball on the ground and throwing it to a base before the runner reaches the base. The defense must record three outs to end the inning.

When a team is on offense, the nine players become batters. Each batter has a turn to go to home plate and face the pitcher. Positioned behind the batter and catcher is an umpire who judges whether pitches are strikes or balls. The batter attempts to get a hit by batting a pitched ball so that it is not caught or he runs to base before a fielder's throw reaches the base. If he gets safely to first base, he has hit a single. Reaching second base safely represents a double, third base a triple, and rounding all of the bases is a home run (usually attained by hitting the ball over the outfield fence.) Every batter who reaches home plate scores a run. Batters have three strikes to put a pitch in play, being called out for swinging and missing on the third strike or “taking” (refraining from swinging at) a pitch that the plate umpire determines was in the strike zone. If the batter refrains from swinging at four pitches that the umpire determines are “balls” outside of the strike zone then the batter earns a base-on-balls, or walk, to first base.
